# The independent and combined effects of blood heavy metal concentrations on all-cause mortality and cardiovascular mortality in adult patients with diabetes mellitus

**DOI:** 10.3389/fpubh.2025.1588078

**Published:** 2025-06-05

**Authors:** Lipeng Cai, Jiangrong Yan, Lei Sun, Weichao Dan

**Affiliations:** ^1^Department of Cardiology, The Third People’s Hospital of Huizhou, The Affiliated Hospital of Guangzhou Medical University, Huizhou, China; ^2^Department of Transfusion, The Third People’s Hospital of Huizhou, The Affiliated Hospital of Guangzhou Medical University, Huizhou, China; ^3^Cardiovascular Department, Huizhou Affiliated Hospital of Sun Yat-sen University, Huizhou, China; ^4^Orthopedic Department, Guoyao North Hospital, Baotou, China

**Keywords:** heavy metals in blood, diabetes mellitus, all-cause mortality, cardiovascular mortality, environmental health

## Abstract

**Background:**

Most epidemiological studies have focused on the association between single metal exposure and cardiovascular disease risk, utilizing a single-pollutant model for analysis. However, multiple metals may interact with each other, leading to misjudgment of health risks. This study sought to ascertain both the independent and combined effects of various blood heavy metal concentrations on all-cause mortality and cardiovascular mortality in patients with DM.

**Methods:**

Patients (≥20 years) with DM from the NHANES (2011–2018) were selected. To explore the relationships of exposure to individual metals, including cadmium (Cd), mercury (Hg), manganese (Mn), lead (Pb), and selenium (Se), with all-cause mortality and cardiovascular mortality, weighted logistic regression and RCS analysis were leveraged. The WQS model was utilized to estimate the effects of combined blood metal exposures.

**Results:**

1,798 patients with DM were included. In the unadjusted model, ln-transformed blood Pb level (OR = 2.3, 95% CI: 1.70–3.10, *p* < 0.001) and ln-transformed Cd level (OR = 1.54, 95% CI: 1.27–1.87, *p* < 0.001) demonstrated positive associations with the all-cause mortality risk. According to RCS analysis, a nonlinear dose–response relationship was noted between Pb, Cd, Se, and the all-cause mortality risk (p-nonlinear < 0.05), while Hg and Mn showed linear relationships (p-nonlinear > 0.05).

**Conclusion:**

According to this study, a high blood concentration of a combination of heavy metals is a significant risk factor for both cardiovascular disease and all-cause mortality of patients with diabetes, with Pb contributing a relatively higher proportion to these risks.

## 1 Introduction

Diabetes mellitus (DM), a metabolic condition, is defined by elevated fasting blood glucose levels, insufficient insulin levels relative to body requirements, or insensitivity to insulin receptors. It is a primary contributor to mortality from cardiovascular disease and all-cause mortality in humans (1). During the past few years, the prevalence of DM has risen rapidly, indicating a high rise in the incidence and death from related diseases in the future (2–6). Extensive research has shown that DM is linked to an enhanced risk of mortality from various causes, including cancer (7, 8). Additionally, prediabetes has been associated with chronic kidney disease, cancer, and all-cause mortality (9–11). Presently, over 450 million persons globally are afflicted with DM, a figure anticipated to escalate to 693 million by 2052. The high morbidity of DM places a heavy burden on both patients and society. The World Health Organization forecasts that it will emerge as the seventh largest cause of death by 2030 (12).

The swift advances in industrialization and urbanization have contributed to an escalation in the discharge of chemicals into the environment. Supplementary Figure S1 shows the spatial distribution of heavy metals around the United States. These substances not only exist independently but are also widespread in mixtures (13). These substances endanger human and animal health, potentially causing a range of diseases such as cancer, birth defects, immune system issues, cognitive decline, and behavioral abnormalities (14). Heavy metals, in particular, are of concern. While some, such as copper, manganese, iron, and zinc, are essential trace elements for human health (15), excessive intake can lead to toxic effects (16). Heavy metal pollution, especially from lead (Pb), mercury (Hg), cadmium (Cd), and arsenic (As), has serious implications for human health as their environmental concentrations continue to rise (17). It is imperative to deeply investigate their complex health impacts.

Population-based research has established correlations between the concentration of a single heavy metal and mortality (18). Moreover, exposure to heavy metal mixtures has been associated with heightened all-cause mortality (18). In the general population, meta-analyses disclosed a strong correlation between exposure to Cd and Pb and all-cause mortality, cardiovascular disease, and cancer (18). Evidence also indicated that exposure to As is related to mortality, although the detrimental effects of Hg and other heavy metals on mortality remain inconclusive (19). Some studies have indicated that the accumulation of heavy metals can induce damage to various organs, leading to chronic kidney disease (20), neurodevelopmental disorders (21), cardiovascular diseases, neuronal injury, diabetes, and cancer (22). However, currently. Currently, epidemiological evidence on the combined effects of exposure to heavy metals on mortality remains scarce.

Humans can simultaneously be exposed to multiple heavy metals, and interactions between these metals may result in synergistic or antagonistic effects. As a result, studies focusing on the impact of single heavy metals on cardiovascular mortality and all-cause mortality may struggle to fully explain the combined effects of heavy metal mixtures. This is because interactions between different heavy metals may involve additive, synergistic, or antagonistic effects, and existing data are often contradictory (23). Furthermore, most current studies primarily focus on the influence of single heavy metal concentrations in the blood on cardiovascular mortality and all-cause mortality. Whether mixtures of heavy metals in blood could increase such risks through certain potential mechanisms, particularly in patients with DM, remains ambiguous. To date, research on the impact of blood heavy metal concentrations on cardiovascular mortality and all-cause mortality in diabetic populations is lacking. Our study endeavored to thoroughly examine the effects of simultaneous exposure to various heavy metals in blood (Cd, Hg, manganese [Mn], Pb, selenium [Se]) on cardiovascular mortality and overall mortality in individuals with DM, with the objective of identifying critical metals of concern. By doing so, we seek to draw more reliable conclusions and provide theoretical insights into the health impacts of heavy metals on humans.

## 2 Methods

### 2.1 Data collection

#### 2.1.1 Database overview

Data from the NHANES were derived for analysis, which was conducted under the approval of the Ethics Review Board of the National Center for Health Statistics. Participants have given their written informed consent before being surveyed (2017) (24). As a nationally representative cross-sectional survey, NHANES utilizes a multistage, stratified, random sampling technique. Since 1999, the study has been undertaken every 2 years to appraise the health and nutritional condition of adults and children in the U. S. (25). The survey combines household interviews with physical examinations in mobile examination centers, including medical, dental, physiological, and laboratory tests. Participant answers questions regarding health, diet, demographics, and socioeconomic status. Each survey cycle includes approximately 10,000 participants, selected through a four-stage stratified cluster sampling process: primary sampling units, census tracts, housing units, and individuals within households (26).

#### 2.1.2 Study population included in the database

Participants aged 20 years and older were collected from the NHANES database between 2011 and 2018. In this study, individuals were defined as having diabetes mellitus if they met any of the following criteria: taking oral hypoglycemic agents or insulin; having a fasting plasma glucose (FPG) level ≥ 126 mg/dL (7.0 mmol/L); or having a HbA1c level ≥ 6.5% (48 mmol/mol). Based on these criteria, a total of 3,036 diabetic patients were initially identified. The following exclusion criteria were applied: (1) 40 participants with missing mortality data or lost to follow-up were excluded; (2) 1,161 participants with missing blood heavy metal levels were excluded; and (3) 291 participants with missing covariates were excluded. Ultimately, 1,544 individuals were included in our analysis.

#### 2.1.3 Assessment of heavy metals in blood

Blood heavy metal exposure was primarily assessed for five metals: Cd, Hg, Mn Pb, and Se. The concentrations of these metals in whole blood samples were measured via mass spectrometry. Following collection, processing, and storage, blood samples were dispatched to the Division of Laboratory Sciences at the National Center for Environmental Health (NCEH) and the Centers for Disease Control and Prevention (CDC) for examination. Comprehensive protocols for sample collection and management are described in the NHANES Laboratory/Medical Technician Procedure Manual. Blood samples were preserved at −30°C until their transfer to the NCEH for analysis.

Via inductively coupled plasma mass spectrometry (ICP-MS), the Cd, total Hg (THg), Mn, Pb, and Se concentrations in the whole blood were measured. Based on quadrupole ICP-MS, this multi-element analytical method generates plasma by coupling radiofrequency power to a moving stream of argon. Following dilution, whole blood samples were aerosolized using a nebulizer, and a portion of the aerosol was atomized and ionized at high temperatures in the plasma. The ions were then transferred into the mass spectrometer through an interface and detected after passing through ion optics and a quadrupole mass analyzer. The ion detection signal was processed into digital information, providing quantitative concentrations for each element. Blood samples were diluted in a 1:1:48 ratio using 18 M-ohm water and a multi-component diluent. Gold (Au) was incorporated to mitigate Hg memory effects, rhodium (Rh) was used as the internal standard for cadmium, and bismuth (Bi) was the internal standard for Hg and Pb. The instruments, laboratory methods, and locations remained unchanged compared to the previous 2 years of NHANES, with Se and Mn measurements being introduced in 2011.

#### 2.1.4 All-cause mortality and cardiovascular mortality in patients with DM

To ascertain the cause of death and mortality status up until the follow-up period’s conclusion on December 31, 2019, mortality data were connected to the National Death Index Public Access Files. The International Classification of Diseases, Tenth Revision (ICD-10), was utilized to code the causes of death. Deaths from cardiovascular disease and deaths from all causes were the primary research outcomes. During the follow-up period, death from any cause was considered all-cause mortality. Mortality from cardiovascular disease encompassed fatalities resulting from heart diseases (ICD-10 codes: I00-I09, I11, I13, I20-I51) and cerebrovascular diseases (ICD-10 codes: I60-I69).

#### 2.1.5 Assessment of other covariates

Demographics collected from participants encompassed age, sex (being male or female), race/ethnicity (Mexican American, other Hispanic, non-Hispanic White, non-Hispanic Black, or others), and education (<9th grade; 9th-11th grade, including 12th grade without a diploma; high school graduate/GED or equivalent; some college or an Associate’s degree; or a college degree or higher). By dividing household income by the poverty threshold that is particular to the size, year, and state of the household, the poverty income ratio (PIR) was determined. PIR was classified into two categories for analysis: low income (poverty) and middle to high-income. Lifestyle factors were extracted from questionnaire responses. The interview question “Have you smoked at least 100 cigarettes in your lifetime?” was used to evaluate smoking status. Individuals who responded “No” were categorized as “non-smokers” and those who responded “Yes” were categorized as “smokers.” Alcohol consumption was classified according to the average daily intake of beverages during the preceding 12 months. One standard drink is quantified as 12 oz. of beer, or 1.5 oz. of distilled spirits, or 5 oz. of wine. Participants were grouped into four categories: less than 5 drinks/d, 5–10 drinks/d, more than 10 drinks/d, and unclear alcohol consumption. Physical activity was categorized based on participants’ responses to whether they engaged in vigorous physical activity—characterized as any physical exercise, fitness regimen, or recreational pursuit that significantly elevates respiration or heart rate, including jogging or basketball, sustained for a minimum duration of 10 min—or moderate recreational activities, which encompass workouts that induce small elevations in respiration or heart rate, including brisk walking, cycling, volleyball or swimming, for a minimum duration of 10 min. Participants were categorized as physically active if they reported engaging in any such activity (≥1 time per week) or as inactive if they did not (<1 time per week). Body mass index (BMI, kg/m^2^) was determined by dividing weight (kg) by height squared (m^2^). Hypertension was classified according to self-reported data, specifically whether a physician or other healthcare professional had ever diagnosed the participant with high blood pressure. Hypercholesterolemia was characterized by whether a physician or other healthcare provider had ever notified the participant of elevated cholesterol levels. Serum total cholesterol level and direct high-density lipoprotein cholesterol (HDL-C) level were measured in the laboratory and included in the study as continuous covariates.

### 2.2 Statistical analysis methods

For all statistical studies, R (v4.4.0) was utilized. The Shapiro–Wilk test was employed to determine if the continuous variables were normal. If the distribution was non-normal, the data would be described with the median and interquartile range (IQR), while normally distributed data is presented as frequency and mean ± standard deviation (SD). Counts and percentages were adopted to present categorical variables. Blood heavy metal concentrations were ln-transformed to correct for skewed distributions. Pearson correlation analysis was employed to assess the relationships among the five heavy metals. In addition, variance inflation factor analysis indicated no multicollinearity among the five heavy metals and covariates, with all VIF values less than 5. Multivariate logistic regression models were employed to calculate the odds ratios (ORs) and their 95% confidence intervals (CIs) for the associations between blood heavy metal levels and all-cause and cardiovascular mortality risks in patients with DM. Then, the relationship between single blood heavy metal concentrations and all-cause and cardiovascular mortality risks in affected patients was evaluated. Model 1 made no covariate adjustments, Model 2 was adjusted regarding sex, age, BMI, race/ethnicity, educational level, and the PIR, while Model 3, alongside the variables included in Model 2, was adjusted for smoking status, alcohol consumption, hypertension, cholesterol, direct HDL-C, and physical activity. Restricted cubic splines and dose–response curves were constructed to evaluate the associations between blood heavy metal levels and the risks of all-cause mortality and cardio-cerebrovascular mortality among diabetic patients. Four knots were selected at 0.05, 0.35, 0.65, and 0.95. Weighted quantile sum (WQS) regression was then employed to investigate the associations between combined blood heavy metal exposure and all-cause and cardiovascular mortality risks. This method further analyzed the combined effect of changes in quantiles within the exposure mixture and calculated the relative contribution (weights) of each exposure. *P* (2-sided) of < 0.05 was deemed statistically significant.

## 3 Results

### 3.1 Baseline characteristics of participants

Table 1 summarizes the characteristics of the included DM patients, categorized by all-cause mortality (characteristics categorized by cardiovascular mortality are presented in Table 2). We included a total of 1,544 patients with DM in this study.

**Table 1:** Population characteristics of all-cause death research, NHANES 2011–2018.

Characteristic	*N* ^1^	Overall, *N* = 1,544	0, *N* = 1,298	1, *N* = 246	*p*-value^3^
Avg # alcohol drinks/day - past 12 mos	1,544				0.002
Drink heavily (>10)		19 (1.0%)	18 (1.2%)	1 (0.2%)	
Drink a little (1 to 5)		717 (54%)	637 (56%)	80 (42%)	
Moderate drinking (6 to 10)		47 (3.3%)	42 (3.6%)	5 (1.4%)	
Unknow		761 (41%)	601 (39%)	160 (56%)	
Gender	1,544				0.262
Men		820 (54%)	669 (55%)	151 (49%)	
Women		724 (46%)	629 (45%)	95 (51%)	
Race	1,544				<0.001
Mexican American		237 (9.7%)	212 (10%)	25 (5.5%)	
Non-Hispanic Black		423 (14%)	352 (15%)	71 (13%)	
Non-Hispanic White		494 (60%)	375 (58%)	119 (74%)	
Other Hispanic		179 (6.2%)	162 (6.8%)	17 (3.1%)	
Other Race - Including Multi-Racial		211 (9.5%)	197 (10%)	14 (4.1%)	
Education	1,544				0.089
9–11th grade (Includes 12th grade with no diploma)		232 (13%)	183 (12%)	49 (16%)	
College graduate or above		294 (23%)	263 (25%)	31 (16%)	
High school graduate/GED or equivalent		358 (23%)	306 (23%)	52 (22%)	
Less than 9th grade		258 (10%)	202 (9.3%)	56 (15%)	
Some college or AA degree		402 (31%)	344 (31%)	58 (30%)	
Body Mass Index (kg/m**2)	1,544	32 (28,37)	32 (28,37)	30 (26,36)	0.032
Hypertension	1,544	998 (63%)	808 (60%)	190 (79%)	<0.001
High cholesterol	1,544	919 (62%)	761 (62%)	158 (63%)	0.778
Age (years)	1,544	60 (50,69)	58 (49, 66)	71 (62, 80)	<0.001
Ratio of family income to poverty	1,544	2.39 (1.20, 4.30)	2.57 (1.22, 4.38)	1.88 (1.12, 3.40)	0.04
Direct HDL-Cholesterol (mg/dL)	1,544	44 (38, 53)	44 (38,53)	46 (37, 55)	0.525
Vigorous recreational activities	1,544	138 (9.3%)	132 (11%)	6 (1.8%)	<0.001
Moderate recreational activities	1,544	510 (35%)	462 (38%)	48 (17%)	<0.001
Smoked at least 100 cigarettes in life	1,544	756 (51%)	609 (50%)	147 (59%)	0.039
Total Cholesterol (mg/dL)	1,544	180 (152, 210)	181 (153, 211)	171 (147, 206)	0.392

^1^N not Missing (unweighted); ^2^n (unweighted) (%); Median (25,75%); ^3^chi-squared test with Rao & Scott’s second-order correction; Wilcoxon rank-sum test for complex survey samples.

**Table 2:** Population characteristics of death from cardiovascular and cerebrovascular diseases, NHANES 2011–2018.

Characteristic	*N* ^1^	Overall, *N* = 1,544	0, *N* = 1,449	1, *N* = 95	*p*-value*^3^*
Avg # alcohol drinks/day - past 12 mos	1,544				0.055
Drink heavily (>10)		19 (1.0%)	18 (1.1%)	1 (0.5%)	
Drink a little (1 to 5)		717 (54%)	688 (55%)	29 (41%)	
Moderate drinking (6 to 10)		47 (3.3%)	45 (3.4%)	2 (1.9%)	
Unknow		761 (41%)	698 (41%)	63 (56%)	
RIAGENDR	1,544				0.269
Men		820 (54%)	767 (55%)	53 (45%)	
Women		724 (46%)	682 (45%)	42 (55%)	
Race	1,544				0.22
Mexican American		237 (9.7%)	226 (9.8%)	11 (7.6%)	
Non-Hispanic Black		423 (14%)	393 (14%)	30 (15%)	
Non-Hispanic White		494 (60%)	451 (60%)	43 (70%)	
Other Hispanic		179 (6.2%)	171 (6.3%)	8 (3.7%)	
Other Race - Including Multi-Racial		211 (9.5%)	208 (9.8%)	3 (3.8%)	
Education	1,544				0.043
9–11th grade (Includes 12th grade with no diploma)		232 (13%)	210 (12%)	22 (20%)	
College graduate or above		294 (23%)	285 (24%)	9 (11%)	
High school graduate/GED or equivalent		358 (23%)	338 (23%)	20 (26%)	
Less than 9th grade		258 (10%)	234 (9.9%)	24 (15%)	
Some college or AA degree		402 (31%)	382 (31%)	20 (27%)	
Body Mass Index (kg/m**2)	1,544	32 (28, 37)	32 (28, 37)	31 (28, 39)	0.787
Hypertension	1,544	998 (63%)	926 (62%)	72 (74%)	0.077
High cholesterol	1,544	919 (62%)	852 (61%)	67 (72%)	0.155
Age(years)	1,544	60 (50, 69)	59 (50, 67)	74 (64, 80)	<0.001
Ratio of family income to poverty	1,544	2.39 (1.20, 4.30)	2.48 (1.22, 4.30)	1.60 (0.98, 2.75)	0.016
Direct HDL-Cholesterol (mg/dL)	1,544	44 (38, 53)	44 (38, 53)	46 (36, 54)	0.754
Vigorous recreational activities	1,544	138 (9.3%)	136 (9.7%)	2 (1.8%)	0.01
Moderate recreational activities	1,544	510 (35%)	494 (36%)	16 (9.7%)	<0.001
Smoked at least 100 cigarettes in life	1,544	756 (51%)	709 (51%)	47 (55%)	0.526
Total Cholesterol (mg/dL)	1,544	180 (152, 210)	181 (152, 211)	171 (147, 206)	0.597

^1^*N* not Missing (unweighted); ^2^*n* (unweighted) (%); Median (25,75%); ^3^chi-squared test with Rao & Scott’s second-order correction; Wilcoxon rank-sum test for complex survey samples. ^#^represents the connection symbol, *represents the square unit.

As shown in Table 1, among them, 1,298 patients (84.07%) did not experience all-cause mortality, while 246 patients (15.93%) did. There were 998 participants (63%) with hypertension, of whom 190 (79%) experienced all-cause mortality, and 808 (60%) did not. Additionally, 138 participants (9.3%) engaged in vigorous recreational activities at least once per week, with 6 of these participants (1.8%) experiencing all-cause mortality, and 132 (11%) not. Patients who experienced all-cause mortality had a lower BMI, were older, and had a lower PIR compared to those who did not (*p* < 0.05). No notable variations were detected between the two groups regarding sex, educational level, presence of hypercholesterolemia, direct HDL-C level, or total cholesterol levels (*p* > 0.05).

### 3.2 Distribution of heavy metals in blood

**Figure 1:**
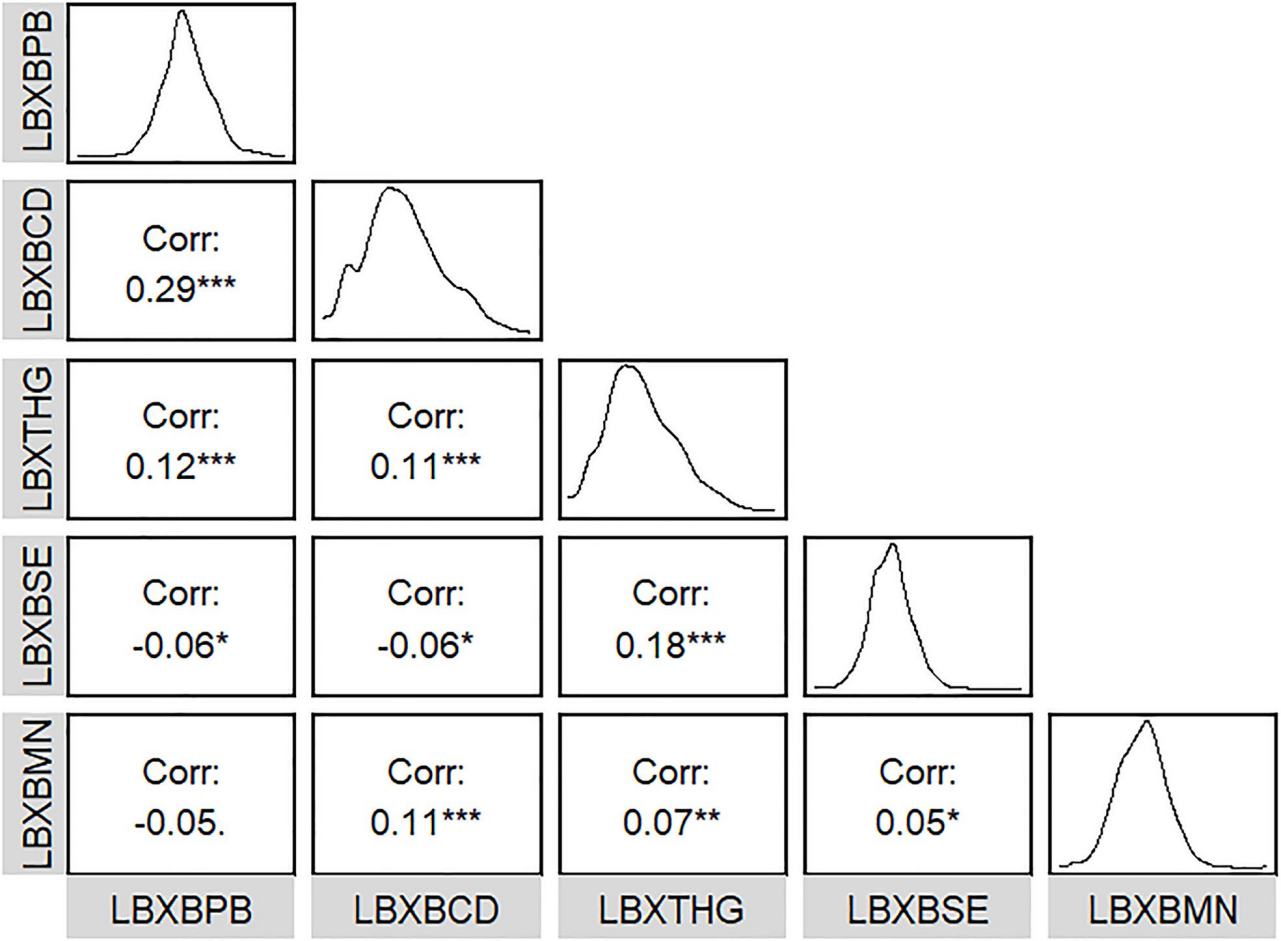
Correlations among five metals in blood.

The detection rates of all five metals in blood exceeded 65.00%. The average ln-transformed blood Pb, Cd, Hg, Se, and Mn levels were 0.14 μg/dL, −1.06 μg/L, −0.14 μg/L, 5.27 μg/L, and 2.2 μg/L, respectively. The relationships between the five metals in blood were weak (all |r| < 0.3) (Figure 1). The distribution of blood heavy metals is illustrated in Supplementary Figure S2.

### 3.3 Association between heavy metals in blood and mortality in patients with DM

The correlation between the ln-transformed concentrations of the investigated heavy metals in blood and the all-cause mortality risk in patients with DM is presented in Table 3. Model 1 made no covariate adjustments, Model 2 was adjusted regarding sex, age, BMI, race/ethnicity, educational level, and the PIR, and Model 3 was adjusted for smoking status, alcohol consumption, hypertension, cholesterol, direct HDL-C, and physical activity, alongside the variables in Model 2. In the unadjusted Model 1, Pb (OR = 2.3, 95% CI = 1.70–3.10, *p* < 0.001), Cd (OR = 1.54, 95% CI = 1.27–1.87, *p* < 0.001), Se (OR = 0.06, 95% CI = 0.02–0.27, *p* < 0.001), and Mn (OR = 0.42, 95% CI = 0.24–0.71, *p* = 0.002) were markedly correlated with all-cause risk mortality in affected patients (*p* < 0.05). No substantial correlation was detected between Hg and the probability of all-cause mortality in these patients (*p* > 0.05). Pb (OR = 1.6, 95% CI = 1.09–2.34, *p* = 0.017) remained remarkably associated with all-cause mortality risk in these patients (*p* < 0.05) after confounders were fully adjusted, while Cd, Hg, Se, and Mn did not (*p* > 0.05). When converting the continuous variables of blood heavy metal levels into categorical variables, we observed in the unadjusted Model 1 that participants in the fourth quartile of blood Pb level (OR = 4.72, 95% CI = 2.61–8.54, *p* < 0.001) and blood Cd level (OR = 2.74, 95% CI = 1.53–4.93, *p* = 0.001) exhibited elevated risks of all-cause mortality relative to those in the first quartile. Conversely, participants in the fourth quartile of blood Se level (OR = 0.52, 95% CI = 0.33–0.81, *p* = 0.005) and blood Mn level (OR = 0.49, 95% CI = 0.27–0.86, *p* = 0.014) had lower all-cause mortality risks, with all showing significant associations with all-cause mortality risk (*p* < 0.05). No substantial correlation was identified between blood Hg level and all-cause mortality risk (*p* > 0.05). After fully adjusting for confounders, participants in the fourth quartile of blood Pb level (OR = 2.12, 95% CI = 1.08–4.19, *p* = 0.032) exhibited an elevated risk of all-cause mortality relative to those in the first quartile. However, Cd (OR = 2.12, 95% CI = 0.70–3.73, *p* = 0.248), Hg (OR = 0.92, 95% CI = 0.54–1.56, *p* = 0.74), Se (OR = 0.68, 95% CI = 0.37–1.24, *p* = 0.203), and Mn (OR = 0.66, 95% CI = 0.36–1.24, *p* = 0.193) showed no significant associations with all-cause mortality (*p* > 0.05).

**Table 3:** Relationship between blood heavy metals and all-cause death in all participants.

		Model 1			Model 2			Model 3		
		OR	95%CI	*p*	OR	95%CI	*p*	OR	95%CI	*p*
Log (Lead)	Continuous	2.3	1.70, 3.10	<0.001	1.69	1.12, 2.53	0.014	1.6	1.09, 2.34	0.017
	Q1	—	—		—	—		—	—	
	Q2	1.41	0.64, 3.11	0.38	0.85	0.38, 1.91	0.68	0.92	0.39, 2.15	0.834
	Q3	2.81	1.36, 5.81	0.006	1.53	0.73, 3.21	0.252	1.55	0.69, 3.46	0.274
	Q4	4.72	2.61, 8.54	<0.001	2.21	1.14, 4.31	0.021	2.12	1.08, 4.19	0.032
	P for trend	<0.001			0.005			0.007		
log (cadmium)	Continuous	1.54	1.27, 1.87	<0.001	1.52	1.17, 1.97	0.003	1.3	0.97, 1.75	0.08
	Q1	—	—		—	—		—	—	
	Q2	1.86	1.08, 3.20	0.025	1.53	0.79, 2.99	0.202	1.4	0.65, 3.00	0.369
	Q3	2.97	1.72, 5.14	<0.001	1.74	0.95, 3.21	0.074	1.54	0.78, 3.03	0.204
	Q4	2.74	1.53, 4.93	0.001	2.23	1.09, 4.58	0.03	1.61	0.70, 3.73	0.248
	P for trend	<0.001			0.02			0.203		
log (mercury)	Continuous	0.87	0.73, 1.04	0.115	0.97	0.78, 1.19	0.739	1.02	0.81, 1.27	0.889
	Q1	—	—		—	—		—	—	
	Q2	1.05	0.59, 1.90	0.856	1.3	0.65, 2.60	0.451	1.45	0.71, 2.95	0.292
	Q3	0.57	0.34, 0.97	0.039	0.64	0.35, 1.17	0.141	0.75	0.38, 1.48	0.386
	Q4	0.65	0.42, 1.02	0.061	0.84	0.49, 1.44	0.517	0.92	0.54, 1.56	0.74
	P for trend	0.011			0.147			0.337		
log (selenium)	Continuous	0.06	0.02, 0.27	<0.001	0.16	0.03, 0.96	0.046	0.17	0.03, 1.11	0.063
	Q1	—	—		—	—		—	—	
	Q2	0.57	0.35, 0.91	0.019	0.57	0.35, 0.95	0.031	0.58	0.34, 0.99	0.046
	Q3	0.45	0.27, 0.76	0.004	0.5	0.28, 0.89	0.019	0.46	0.24, 0.88	0.022
	Q4	0.52	0.33, 0.81	0.005	0.68	0.37, 1.24	0.203	0.72	0.38, 1.34	0.282
	P for trend	0.004			0.166			0.216		
log (manganese)	Continuous	0.42	0.24, 0.71	0.002	0.63	0.38, 1.05	0.074	0.68	0.42, 1.09	0.103
	Q1	—	—		—	—		—	—	
	Q2	0.56	0.33, 0.95	0.033	0.58	0.33, 1.03	0.063	0.63	0.33, 1.21	0.158
	Q3	0.57	0.36, 0.92	0.023	0.66	0.39, 1.13	0.126	0.78	0.44, 1.36	0.36
	Q4	0.49	0.27, 0.86	0.014	0.66	0.36, 1.24	0.193	0.69	0.36, 1.32	0.249
	P for trend	0.012			0.189			0.288		

Model 1 is not adjusted by any covariate; Model 2 adjusts gender, age, body mass index, race, education level, family income and poverty ratio on the basis of model 1; Model 3 adjusted smoking status, drinking status, hypertension, cholesterol, direct high density lipoprotein cholesterol and physical activity on the basis of model 2.

Table 4 illustrates the correlation between the five heavy metals in blood and cardiovascular mortality risk in affected patients. Results demonstrated that when the five heavy metals in blood were analyzed as continuous variables, the unadjusted model revealed significant associations with cardiovascular mortality risk in affected patients for Pb (OR = 2.06, 95% CI = 1.50–2.85, *p* < 0.001), Se (OR = 0.09, 95% CI = 0.01–0.75, *p* = 0.027), and Mn (OR = 0.40, 95% CI = 0.17–0.92, *p* = 0.032) (*p* < 0.05), while Cd was not (*p* > 0.05). Upon complete adjustment for covariates, Pb (OR = 1.47, 95% CI = 1.06–2.02, *p* = 0.021) continued to exhibit a significant association with cardiovascular mortality risk in these patients (*p* < 0.05), while Cd, Hg, Se, and Mn did not (*p* > 0.05). When the continuous variables of blood heavy metal levels were converted into categorical variables, in the unadjusted model, we observed that participants in the fourth quartile of blood Pb level (OR = 5.45, 95% CI = 1.68–17.7, *p* = 0.006) exhibited a significantly elevated risk of mortality from cardiovascular disease in relative to those in the first quartile. Conversely, participants in the fourth quartile of blood Mn level (OR = 0.44, 95% CI = 0.24–0.81, *p* = 0.01) had a significantly lower risk of cardiovascular mortality, with both Pb and Mn being significantly associated with cardiovascular mortality risk (*p* < 0.05). No substantial correlations were identified between Cd, Hg, or Se and cardiovascular mortality risk (*p* > 0.05). Mone of the heavy metals—Pb, Cd, Hg, Se, or Mn—were remarkably associated with cardiovascular mortality when comparing participants in the fourth quartile to those in the first quartile after confounders were fully adjusted (*p* > 0.05).

**Table 4:** Relationship between blood heavy metals and cardiovascular death in all participants.

		Model 1			Model 2			Model 3		
		OR	95%CI	*p*	OR	95%CI	*p*	OR	95%CI	*p*
log (Lead)	Continuous	2.06	1.50, 2.85	<0.001	1.47	0.97, 2.23	0.068	1.47	1.06, 2.02	0.021
	Q1	—	—		—	—		—	—	
	Q2	2.1	0.61, 7.27	0.236	1.38	0.39, 4.87	0.607	1.42	0.41, 4.93	0.566
	Q3	3.64	1.37, 9.65	0.011	2.1	0.79, 5.61	0.133	2.17	0.86, 5.49	0.096
	Q4	5.45	1.68, 17.7	0.006	2.5	0.68, 9.15	0.161	2.48	0.75, 8.22	0.13
	P for trend	<0.001			0.06			0.044		
log (cadmium)	Continuous	1.28	0.96, 1.71	0.091	1.18	0.80, 1.74	0.395	1.09	0.67, 1.78	0.707
	Q1	—	—		—	—		—	—	
	Q2	2.02	0.96, 4.25	0.062	1.57	0.66, 3.69	0.296	1.61	0.64, 4.04	0.292
	Q3	2.12	0.94, 4.82	0.071	1.04	0.43, 2.52	0.924	1.04	0.41, 2.69	0.925
	Q4	1.83	0.73, 4.61	0.195	1.32	0.47, 3.73	0.59	1.19	0.32, 4.36	0.784
	P for trend	0.169			0.824			0.999		
log (mercury)	Continuous	0.74	0.47, 1.17	0.197	0.84	0.52, 1.37	0.48	0.88	0.53, 1.46	0.604
	Q1	—	—		—	—		—	—	
	Q2	0.78	0.29, 2.10	0.612	0.85	0.29, 2.51	0.765	0.85	0.27, 2.72	0.773
	Q3	0.31	0.15, 0.65	0.002	0.33	0.15, 0.71	0.006	0.35	0.15, 0.80	0.015
	Q4	0.49	0.20, 1.21	0.119	0.67	0.25, 1.80	0.41	0.72	0.25, 2.13	0.54
	P for trend	0.036			0.129			0.225		
log (selenium)	Continuous	0.09	0.01, 0.75	0.027	0.34	0.04, 2.80	0.305	0.29	0.03, 2.78	0.268
	Q1	—	—		—	—		—	—	
	Q2	0.65	0.35, 1.19	0.156	0.7	0.39, 1.26	0.228	0.64	0.35, 1.16	0.133
	Q3	0.32	0.16, 0.63	0.002	0.4	0.19, 0.83	0.015	0.35	0.16, 0.76	0.011
	Q4	0.6	0.28, 1.26	0.173	0.89	0.38, 2.06	0.775	0.82	0.34, 1.99	0.649
	P for trend	0.105			0.541			0.456		
log (manganese)	Continuous	0.4	0.17, 0.92	0.032	0.65	0.28, 1.50	0.304	0.73	0.30, 1.77	0.467
	Q1	—	—		—	—		—	—	
	Q2	0.38	0.16, 0.92	0.033	0.4	0.14, 1.17	0.092	0.38	0.12, 1.21	0.096
	Q3	0.46	0.26, 0.83	0.011	0.58	0.28, 1.19	0.131	0.61	0.27, 1.36	0.213
	Q4	0.44	0.24, 0.81	0.01	0.62	0.30, 1.27	0.18	0.65	0.31, 1.37	0.244
	P for trend	0.017			0.225			0.324		

Model 1 is not adjusted by any covariate; Model 2 adjusts gender, age, body mass index, race, education level, family income and poverty ratio on the basis of model 1; Model 3 adjusted smoking status, drinking status, hypertension, cholesterol, direct high density lipoprotein cholesterol and physical activity on the basis of model 2.

### 3.4 Dose–response relationship between heavy metals in blood and all-cause mortality and cardiovascular mortality in patients with DM

The dose–response relationships between blood heavy metal levels and both all-cause mortality and cardiovascular mortality in affected patients were analyzed using the RCS regression model.

**Figure 2:**
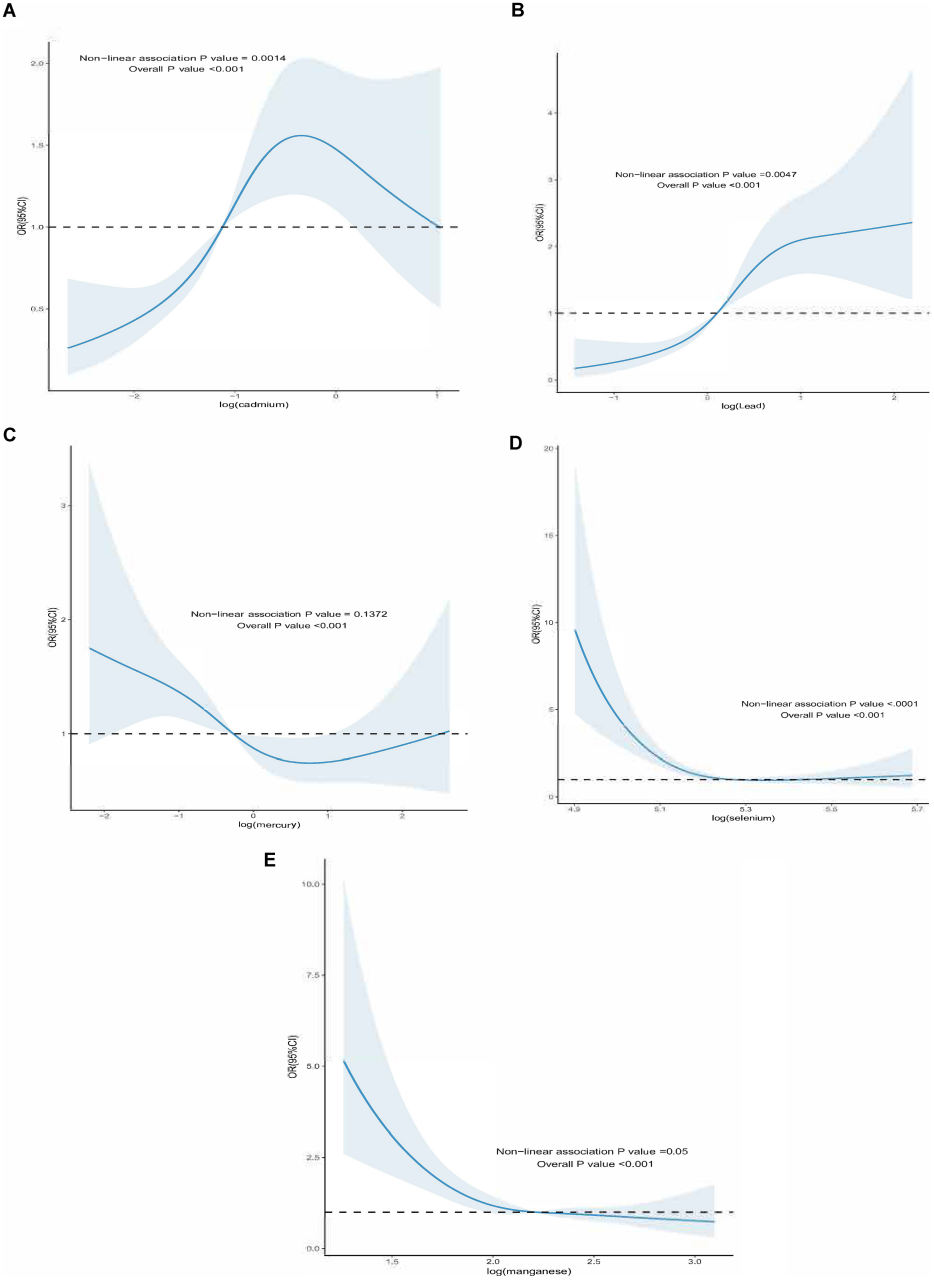
Dose–response effect between blood heavy metal content and all-cause death risk of diabetic patients. **(A)** The dose–response relationship between the lead content in blood and the risk of all-cause death in diabetic patients (fully adjusted RCS model). The baseline value (*β* = 1) is represented by a dotted line, and the inflection point appears at 0.120 (blood lead content after logarithmic conversion). **(B)** the relationship between the cadmium content in blood and the risk of all-cause death of diabetic patients (fully adjusted RCS model), the baseline value (*β* = 1) is represented by a dotted line, and the inflection point appears at −1.118 (logarithmic converted blood cadmium content) **(C)** the relationship between the mercury content in blood and the risk of all-cause death of diabetic patients is linear (fully adjusted RCS model). **(D)** The dose–response relationship between the blood selenium content and the risk of all-cause death of diabetic patients (fully adjusted RCS model), the baseline value (*β* = 1) is represented by a dotted line, and the inflection point appears at 5.265 (blood selenium content after logarithmic conversion). **(E)** The dose–response relationship between the manganese content in blood and the risk of all-cause death in diabetic patients (fully adjusted RCS model). The baseline value (*β* = 1) is indicated by the dotted line, and the inflection point appears at 2.211 (logarithmic converted blood manganese content). The adjustment of the model takes into account the following variables: age, gender, race, education level, exercise, smoking status, BMI, family PIR, drinking status, education level, direct high-density lipoprotein cholesterol, hyperlipidemia and hypert.

**Figure 3:**
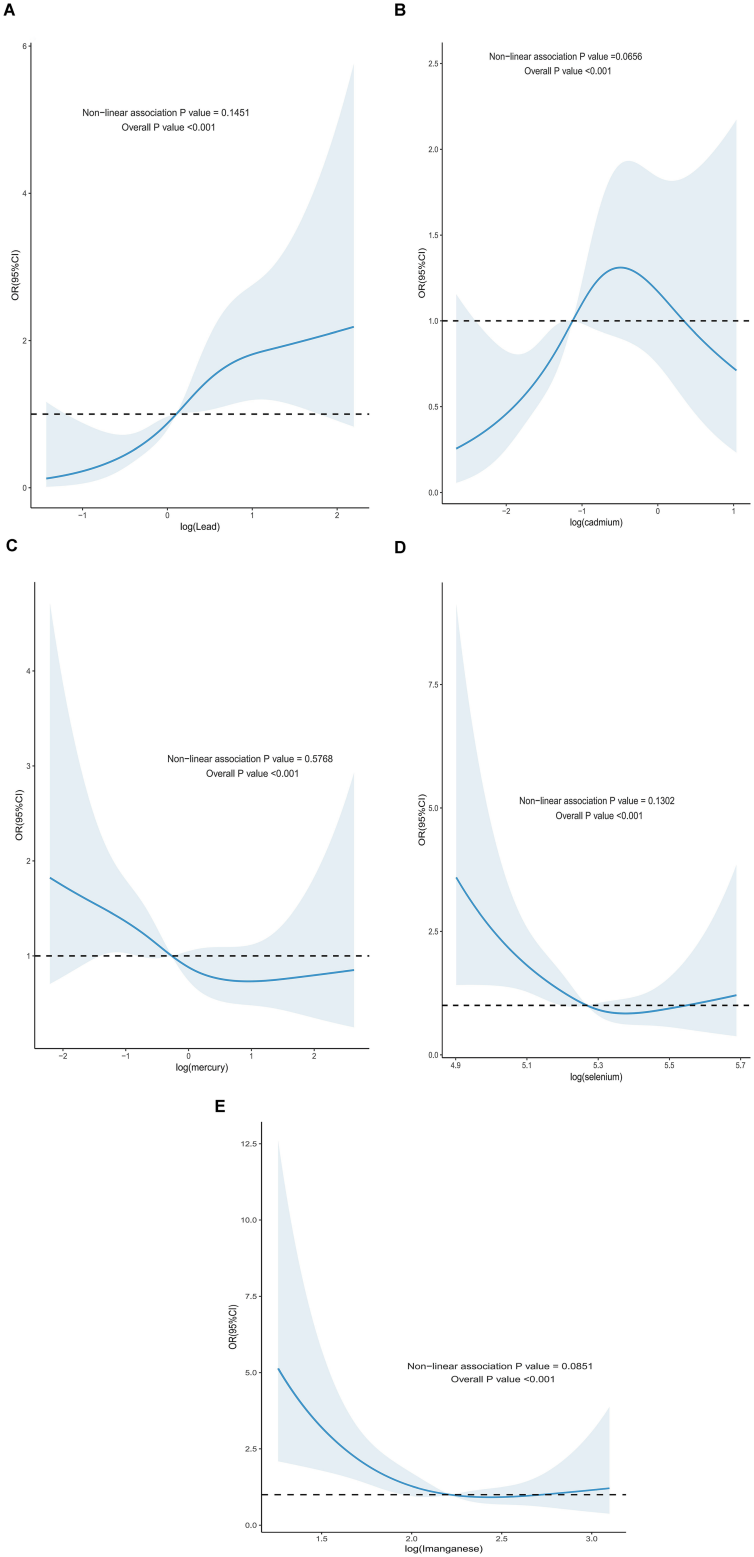
Dose–response effect between blood heavy metal content and risk of all-cause death in diabetic patients. **(A)** There is a linear relationship between the lead content in blood and the risk of all-cause death in diabetic patients (fully adjusted RCS model). **(B)** There is a linear relationship between the concentration of cadmium in blood and the risk of all-cause death in diabetic patients (fully adjusted RCS model). **(C)** There is a linear relationship between the content of mercury in blood and the dose–response between the content and the risk of all-cause death in diabetic patients (fully adjusted RCS model). **(D)** There is a linear relationship between the selenium content in blood and the risk of all-cause death in diabetic patients (fully adjusted RCS model). **(E)** There is a linear relationship between the manganese content in blood and the risk of all-cause death in diabetic patients (fully adjusted RCS model). The adjustment of the model takes into account the following variables: age, gender, race, education level, exercise, smoking status, BMI, family PIR, drinking status, education level, direct high-density lipoprotein cholesterol, hyperlipidemia and hypertension.

For all-cause mortality, the blood ln-transformed levels of Hg and Mn showed a linear dose–response relationship in patients with DM (*p*-nonlinear>0.05, overall *p* value<0.001). In contrast, the blood ln-transformed levels of Pb, Cd, and Se exhibited a non-linear dose–response relationship in these patients (*p*-nonlinear<0.05, overall *p* value<0.001). For all-cause mortality in affected patients, the inflection points for blood ln-transformed heavy metal levels were identified as follows: ln-Pb at 0.120, ln-Cd at −1.118, and ln-Se at 5.265 (Figure 2). Regarding cardiovascular mortality, the dose–response relationships between blood ln-transformed Pb, Cd, Hg, Se, and Mn levels and cardiovascular mortality risk in affected patients were linear (*p*-nonlinear > 0.05, overall *p* value<0.001) (Figure 3).

**Figure 4:**
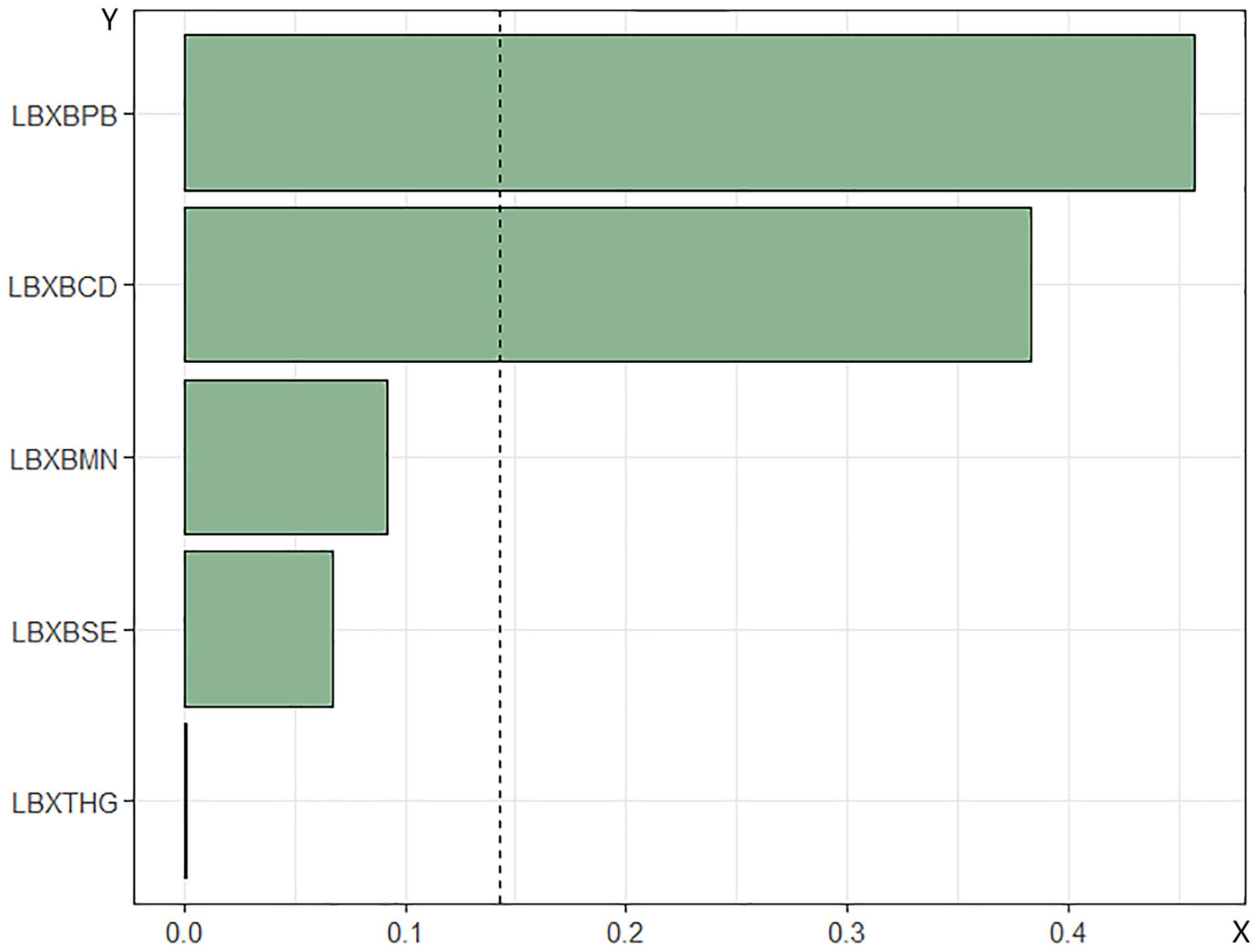
Relationship between WQS index of blood heavy metal mixture and risk of all-cause death in diabetic patients. Lbxpb-lead; Lxbcd-cadmium; LBXBMN- manganese; Lxbse-selenium; Lbxhg-mercury. The adjustment of the whole model takes into account the following variables: age, gender, race, education level, exercise, smoking status, BMI, family PIR, drinking status, education level and direct high density lipoprotein. Cholesterol, hyperlipidemia and hypertension.

**Figure 5:**
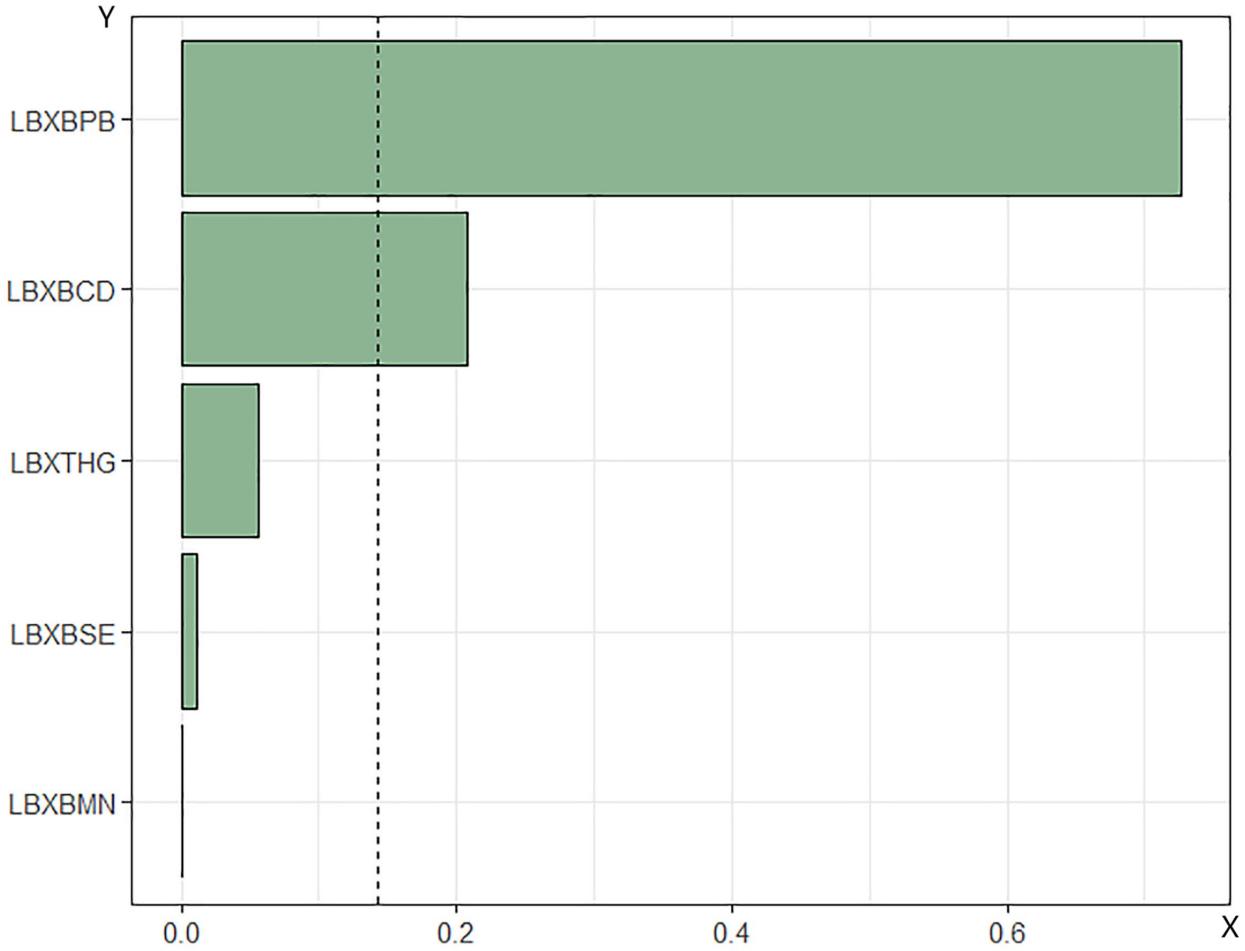
Relationship between WQS index of blood heavy metal mixture and death risk of cardiovascular and cerebrovascular diseases in diabetic patients. Lbxpb-lead; Lxbcd-cadmium; LBXBMN- manganese; Lxbse-selenium; Lbxhg-mercury. The adjustment of the whole model takes into account the following variables: age, gender, race, education level, exercise, smoking status, BMI, family PIR, drinking status, education level, straight. High density lipoprotein cholesterol, hyperlipidemia and hypertension.

WQS regression analysis of metal mixtures in blood and mortality in patients with DM. The combined effect of the mixture of heavy metals in blood on mortality risk in patients with DM was assessed using WQS regression. As shown in Figures 4, 5, the WQS index for heavy metal mixtures in blood exhibited a substantial positive correlation with all-cause mortality risk in patients (OR = 1.68, 95% CI = 1.22–2.30, *p* = 0.001), with Pb being the predominant contributor to this association with mortality risk (45.76%). Similarly, the WQS index for heavy metal mixtures in the blood significantly positively associated with cardiovascular mortality risk in these patients (OR = 1.71, 95% CI = 1.15–2.55, *p* = 0.008), with Pb again having the highest weight in the association (72.59%).

## 4 Discussion

Through this study, it was identified that the risk of all-cause mortality in patients with DM was strongly correlated with blood ln-transformed Pb, Cd, Se, and Mn levels. After adjusting for multiple confounders, Pb was the sole heavy metal significantly correlated with all-cause mortality risk. Moreover, elevated blood Pb level was correlated with an enhanced cardiovascular mortality risk. Furthermore, the heavy metal mixtures in blood were significantly positively associated with both all-cause mortality and cardiovascular mortality risks in these patients, with Pb contributing the most to these associations. For all-cause mortality risk, controlling the Ln Pb concentration in the body to less than 0.12, the Ln Cd concentration to greater than −1.118, and the Ln Se concentration to greater than 5.265 can reduce the all-cause mortality risk in diabetic patients. These findings underscored the potential impact of exposure to Pb on mortality risk in patients with DM and suggested the importance of reducing heavy metal exposure to improve prognosis in this population.

With the global burden of DM rising rapidly, it poses a significant challenge to socioeconomic systems and human health. By 2040, the estimated number of individuals with DM will reach 642 million, a significant increase from the 415 million recorded in 2015 (27), available online: http://www.who.int/diabetes/global-report/en/ (28). A 2015 narrative study noted that exposure to environmental chemicals and polluted air may promote the development of type 1 DM by damaging immune cells or *β*-cell function (29). Comprehensive strategies for DM prevention must include interventions for improving environmental factors. Consequently, it is imperative to examine the effects of environmental metals on patients with DM.

Heavy metals accumulate primarily in the liver, kidneys, and pancreas, where they alter and impair the activity of key enzymes, leading to detrimental effects on glucose metabolism and associated metabolic pathways. Disruption of hepatic glucose homeostasis is crucial in the development of DM. Damage to liver and kidney function, as well as reduced pancreatic and muscle function, can significantly elevate blood glucose levels, ultimately triggering DM. While the development of DM from exposure to a single chemical is exceedingly rare (30), the more common scenario involves the combined exposure to multiple chemicals along with other risk factors, which together contribute to the onset of DM (31). Common heavy metals such as Cd, Hg, Mn, Pb, and Se have drawn considerable interest due to their global prevalence and recognized detrimental health consequences at elevated concentrations. Previous research has demonstrated that even at lower exposure levels, Cd remains a significant determinant of all-cause mortality and cardiovascular mortality among U. S. adults (32). Elevated serum Mn concentration has also been linked to all-cause mortality and cardiovascular mortality (33). Additionally, exposure to Pb and Cd, either individually or in combination, significantly increases the mortality risk in patients with T2DM (34). Our findings align with existing studies, indicating that elevated levels of Cd and Pb in the body correlate with increased risks of all-cause, cardiovascular, and cancer-related death. The study showed that, after controlling for confounding factors, patients with high Pb levels had a significantly increased risk of dying from cardiovascular disease compared to those with low Pb levels. In other words, an increase in blood Pb concentration is associated with an increased risk of cardiovascular mortality (35). However, these prior studies did not explore the combined effects of Cd, Hg, Mn, Pb, and Se on mortality outcomes, nor did they specifically focus on the diabetic population. Building on these previous studies, our research not only examined the impact of single heavy metals in blood on patients with DM but also, for the first time, incorporated the WQS regression model to assess the combined effects of the five heavy metals on mortality risk in these patients. This innovative approach provided clear insights into both the single and combined effects of these metals on mortality outcomes in this vulnerable population.

Although the underlying mechanisms linking blood heavy metal levels with heightened all-cause and cardiovascular mortality risk in these patients remain to be fully elucidated, evidence has indicated that exposure to heavy metals may induce oxidative stress, leading to DNA damage, protein modifications, and lipid peroxidation. These processes can result in various health issues, including cardiovascular disease, neurotoxicity, kidney damage, and elevated risks of cancer, DM, and infertility (36). Therefore, more mechanistic research is required to clarify the possible mechanisms by which blood heavy metal levels in DM patients serve as predictors of mortality risk from cardiovascular diseases and also all causes.

Heavy metals in blood are ubiquitous in our environment, and understanding their potential health impacts is crucial. This study has several strengths: we identified associations between blood heavy metal levels and the risks of all-cause and cardiovascular mortality in patients with DM using a nationally representative dataset, which enhances the accuracy of our findings and facilitates the controlling of potential confounding variables. Additionally, utilizing NHANES data along with suitable weighting methodologies allows for the generalization of results to the non-institutionalized adult populations of Hispanic, White, and Black Americans. Moreover, while variability in blood heavy metal concentrations may arise from alterations in diet, daily activities, or personal care products, we focused on concentrations above the detection limit, ensuring that the blood heavy metal levels were accurately and precisely quantified.

However, our study has some limitations. First, blood heavy metal concentrations were detected at a single time point, and since these levels can fluctuate over relatively short periods, they may not correctly reflect an individual’s usual exposure. Furthermore, it is important to note that the NHANES database is essentially a cross-sectional study database, where each participant contributes only one set of measurements. Therefore, there is no method within the database to achieve temporal stability through repeated measurements. Secondly, although the inclusion and exclusion criteria in this study were designed to focus on the diabetic patient population to ensure consistency of the study group and accuracy of data analysis, these criteria may limit the generalizability of the study results. For instance, excluding non-diabetic patients means that the findings are primarily applicable to the diabetic population and cannot be directly generalized to a broader population. Future studies should consider optimizing the inclusion and exclusion criteria to enhance the representativeness and generalizability of the results. Furthermore, the potential for residual confounding arising from psychosocial variables, genetic predisposition, unidentified confounders, or random variation cannot be completely excluded. Last, further experimental investigations are required to elucidate the mechanisms by which blood heavy metal levels influence all-cause and cardiovascular mortality risks in individuals with DM.

## 5 Conclusion

Our study innovatively incorporated the WQS model to assess heavy metals in blood, and the results indicated that mixed heavy metals in blood are strong contributors to all-cause and cardiovascular mortality risks in patients with DM. Reducing Pb exposure may have potential benefits in decreasing the premature death risk among affected patients.

## Data Availability

The original contributions presented in the study are included in the article/Supplementary material, further inquiries can be directed to the corresponding author.

## Supplementary material

SUPPLEMENTARY FIGURE S1 The spatial distribution of heavy metals around the United States.

SUPPLEMENTARY FIGURE S2 Distribution histogram of five kinds of blood heavy metals
