# Supplementary figures and images for: The independent and combined effects of blood heavy metal concentrations on all-cause mortality and cardiovascular mortality in adult patients with diabetes mellitus
Source: Front Public Health. 2025 Jun 5;13:1588078. doi: 10.3389/fpubh.2025.1588078 (PMC12176894; doi:10.3389/fpubh.2025.1588078)

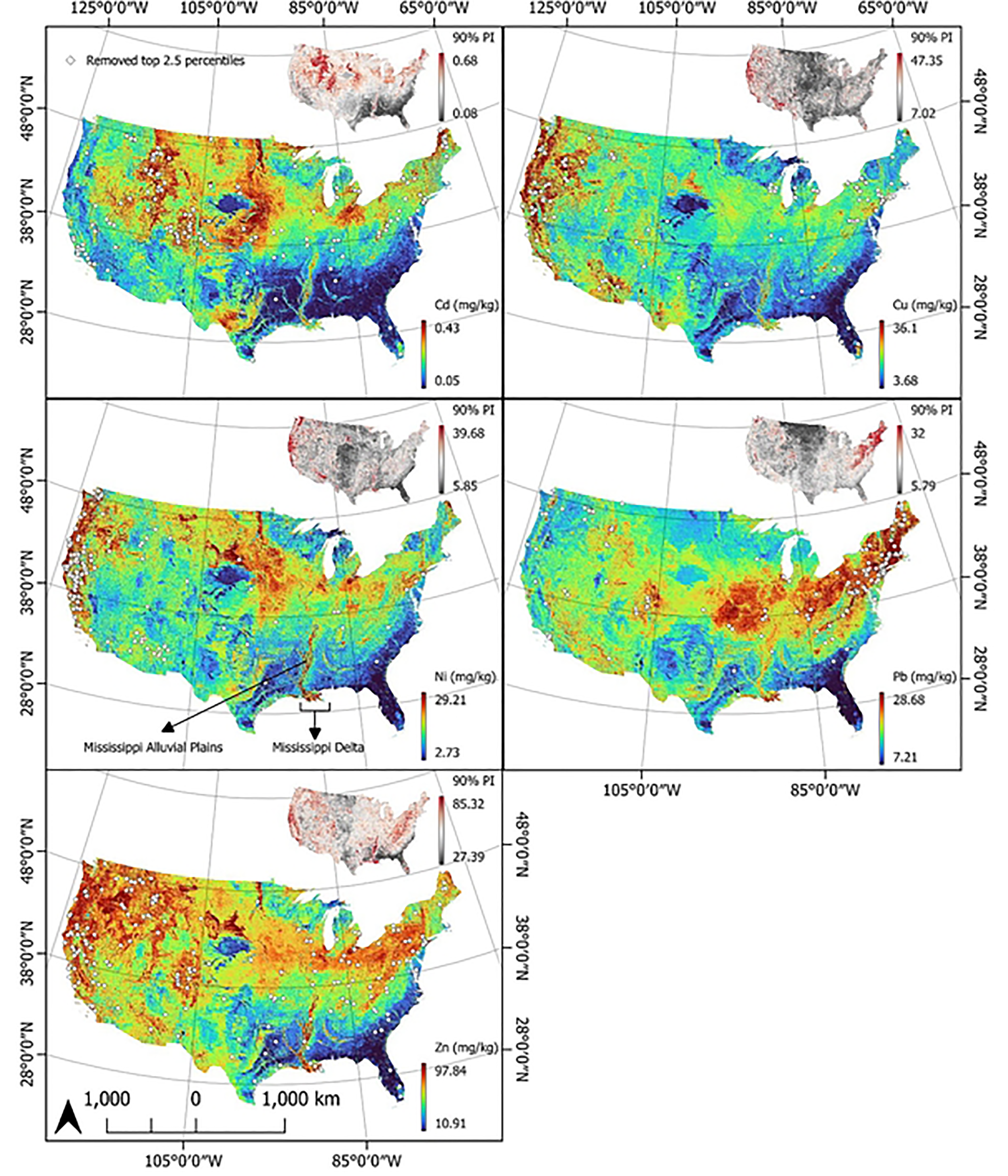

Supplement: SUPPLEMENTARY FIGURE S1 — The spatial distribution of heavy metals around the United States. [file Image_1.tif]

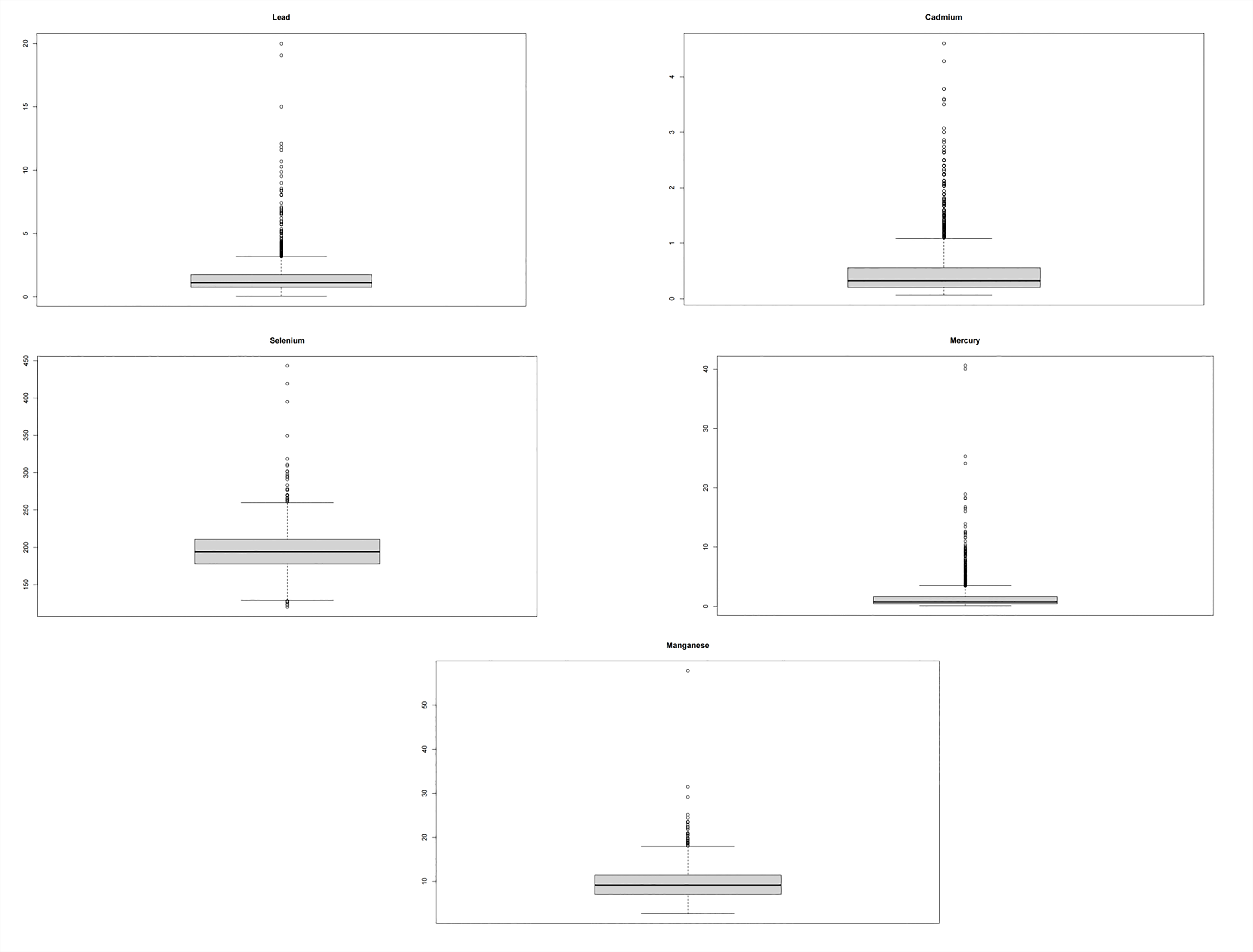

Supplement: SUPPLEMENTARY FIGURE S2 — Distribution histogram of five kinds of blood heavy metals [file Image_2.tif]
